# Association between fibrinogen concentration and nonunion in fracture patients

**DOI:** 10.3389/fsurg.2026.1607136

**Published:** 2026-03-06

**Authors:** Lili Geng, Zejun Wang, Jinlei Dong, Bingru Lu, Jincan Wang, Yuqin Wang, Yiqing Liu

**Affiliations:** 1 Department of Clinical Laboratory, Shandong Provincial Hospital Affiliated to Shandong First Medical University, Jinan, Shandong, China; 2 Department of Orthopaedics Surgery, Shandong Provincial Hospital Affiliated to Shandong First Medical University, Jinan, Shandong, China; 3 School of Public Health, Shandong First Medical University, Jinan, Shandong, China

**Keywords:** fracture, nonunion, open reduction and internal fixation (ORIF), plasma fibrinogen, retrospective analysis

## Abstract

Nonunion (non-osteogenic healing) remains a major challenge in fracture management, particularly due to its diagnostic complexity and unpredictable occurrence in clavicle and femoral fractures. This study aimed to investigate the potential association between plasma fibrinogen concentration and the incidence of nonunion in fracture patients, with the objective of identifying a potential biomarker for clinical prediction. Based on retrospective data from Shandong Provincial Hospital Affiliated to Shandong First Medical University (January 2010 to May 2019), we analyzed a cohort of 338 fracture cases, among which 23 (6.8%) developed nonunion. Fibrinogen concentration (AUC = 0.635, 95% CI 0.517–0.752) showed moderate discriminative capability for nonunion. Using smoothing curve fitting techniques and multivariable logistic regression models, the study systematically assessed the dose-response relationship between fibrinogen levels and the risk of nonunion risk, adjusting for confounding factors such as age, sex, injury mechanism, and ASA classification. The results indicated that for every 1 g/L increase in fibrinogen concentration, the risk of nonunion increased significantly by 48% [adjusted odds ratio [OR] = 1.48, 95% confidence interval [CI] not explicitly reported but implied statistical significance]. This association remained statistically significant even after controlling for traditional risk factors such as trauma severity and baseline patient status, suggesting that fibrinogen may influence bone healing through independent pathways. Smoothing curve fitting revealed a nonlinear, dose-dependent increase in nonunion risk with higher fibrinogen levels, potentially guiding the establishment of clinical threshold settings. The study found that elevated plasma fibrinogen levels are independently associated with an increased risk of nonunion, and routine monitoring of fibrinogen concentrations may serve as a promising adjunct tool for early identification of at-risk patients. Future research should focus on elucidating the underlying mechanisms-such as inflammation regulation and extracellular matrix deposition-and validating the predictive value of fibrinogen across different types of fractures to support the development of personalized treatment strategies.

## Introduction

Nonunion is a medical condition characterized by the failure of a fracture to heal, which can result from various factors such as infection, tumor, trauma, type of surgery, or congenital abnormalities. Normally, bone tissue generally has a strong capability to repair itself, and most fractures heal well with appropriate treatment. However, some fractures either heal slowly (delayed healing) or fail to heal altogether (nonunion). According to the most consistent standard definition provided by the Food and Drug Administration(FDA)**,** nonunion occurs when a fracture remains unhealed at least nine months without showing progressive signs of healing over a three-month period (1–3). Studies have found that about 5%–20% of patients with fractures experience delayed healing or nonunion (4), significantly impairing their quality of life. Our previous studies showed that there are many factors affecting nonunion, for instance, tobacco has a negative effect on fracture healing, leading to delayed bone healing and increased probability of nonunion in fracture patients (5). The rising incidence of fractures among the elderly populations may reflect mutiple concurrent trends. For example, the prevalence of fractures increases significantly with age (6). Age-related conditions such as diabetes mellitus (7), stroke (8), and Parkinson's disease (9) also elevate the risk of falls and impair fracture healing. Furthermore, evidence also demonstrates that certain medications, particularly glucocorticoids, can affect fracture healing, ultimately increasing the risk of nonunion (10). A moderate and controlled acute inflammatory response in the early stage (0–72 h) is the “decisive” factor for the successful initiation of bone regeneration, blocking or prolonging this phase leads to failure in bone healing (11–13).Using smoothing curve fitting techniques and multivariable logistic regression models, we found that higher fibrinogen levels were significantly associated with an increased risk of non-healing, though causality cannot be established from observational data.

Accurate diagnosis of nonunion is critical for effective clinical management and treatment planning of fractures. However, there is a lack of effective tools to evaluate fracture healing status. Therefore, it is very important to identify the diagnostic indicators of nonunion. Serological examination is a commonly used method in clinical diagnostics. Previous studies have shown that monocytes participate in angiogenesis and differentiate into osteoclasts during fracture healing. Many bone morphogenesis-related protein changes occurred in human peripheral blood mononuclear cells after fracture, suggesting that gene changes in human peripheral blood mononuclear cells may serve as an early predictor of nonunion (14). Other coagulation-related indicators, such as fibrinogen (FIB) and D-dimer, have been proposed as laboratory indicators for the diagnosis of infectious nonunion (15, 16). Previous studies have shown that the intrinsic components of fibrin matrix are essential for initiating bone formation. Given that impaired fibrinolysis is a comorbid disease associated with impaired fracture repair, we hypothesized that although extravascular fibrin matrix shows a beneficial effect on fracture repair, persistent fibrin deposition shows a detrimental effect on fracture repair (17). Therefore,Monocytes may release cytokines at inflammatory sites, which may stimulate the liver to produce more fibrinogen, thereby promoting coagulation. Additionally, fibrinogen may serve as a signaling molecule for monocyte migration or activation, aiding their arrival at damaged tissues. Fibrinogen may be cleaved into fibrinopeptides, which could influence monocyte activity and exacerbate inflammatory responses. When fibrinogen is converted into fibrin, the resulting fibrin mesh not only arrests bleeding but also captures and concentrates monocytes at the injury site, thereby amplifying localized inflammation and facilitating tissue repair. SO it is very important to establish a laboratory diagnostic method for the diagnosis and treatment of nonunion. In this study, we investigated the essential function of fibrinogen on effective fracture repair and the persistent fibrin matrix ultimately impeding the normal fracture repair.

Studies have revealed that the optimal threshold of fibrinogen concentration at 2.75 g/L showed the highest sensitivity and sound specificity to detect the nonunion (16). Furthermore, fibrinogen is believed to play a key role in tissue repair, promoting new bone formation (18). Therefore, this study aimed to investigate the relationship between fibrinogen concentration and nonunion in the fracture patients collected at the Shandong Provincial Hospital Affiliated to Shandong First Medical University from January 2010 to May 2019.

## Materials and methods

### Study objects

**Figure 1:**
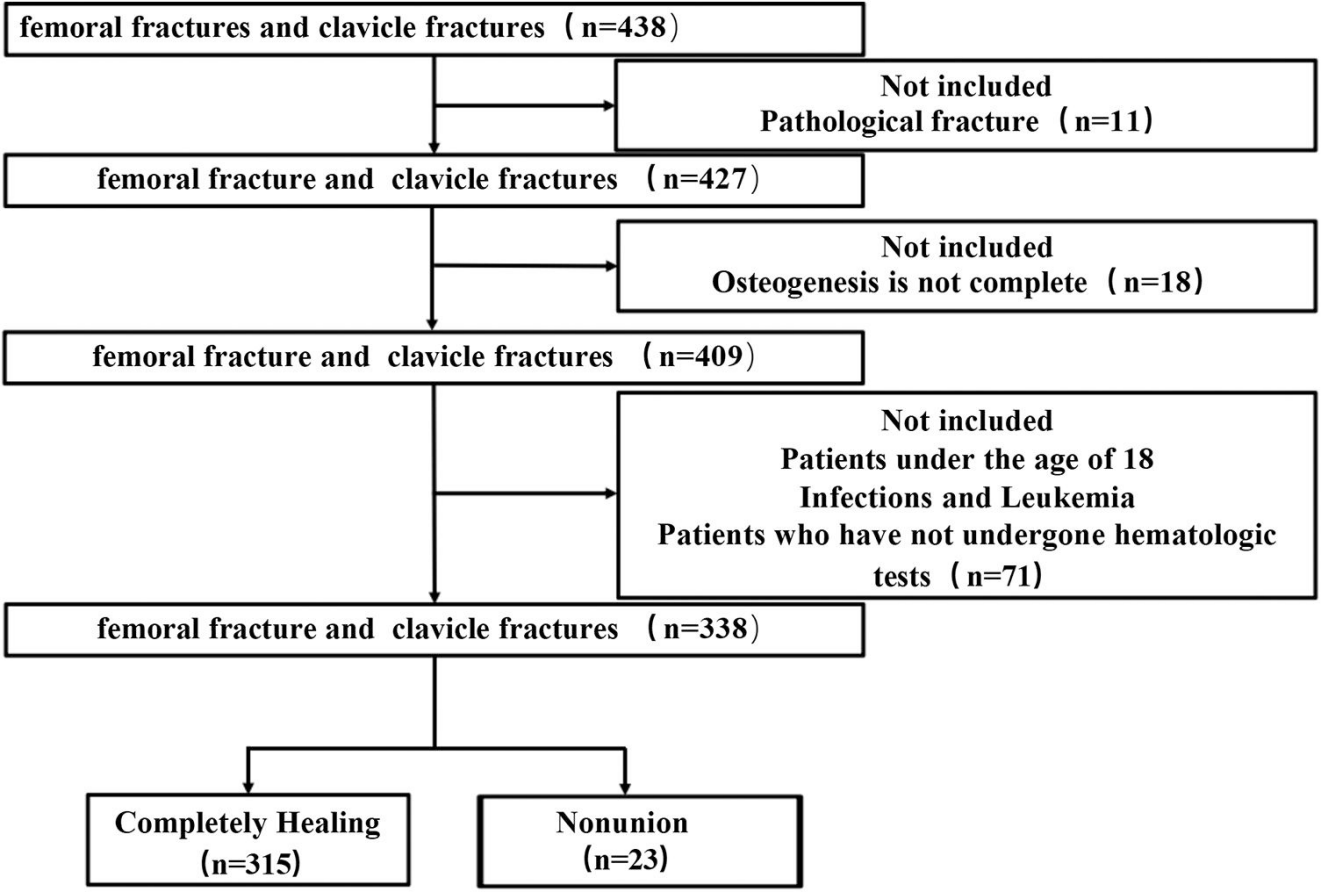
The selection criteria of patients of fracture in this study.

We conducted a retrospective analysis of 438 patients with either clavicle or femur fractures at the Shandong Provincial Hospital Affiliated to Shandong First Medical University from January 2010 to May 2019. Patients were excluded based on discharge criteria, including pathological fractures (11 cases) and osteogenic insufficiency referred to patients with metabolic bone diseases (e.g., osteoporosis) or osteomalacia (18 cases), patients under 18 years of age, with infections and leukemia, and lack of laboratory tests (71 cases). A total of 338 patients with fractures (87 femoral fractures and 251 clavicle fractures) were chosen for further analyses (Figure 1). In these cases, 315 patients showed complete healing, while nonunion were observed in 23 patients. The diagnostic criteria of nonunion based on the FDA guidelines (i.e., at least 9 months post-fracture without healing, and no signs of progressive healing for 3 consecutive months), which echoes the standards mentioned in our Introduction (19).The determination of healing is based on the presence of bridging callus on at least three out of four cortices on standard anteroposterior and lateral radiographs, as well as the disappearance of the fracture line. Standard anteroposterior and lateral radiographs were routinely obtained immediately postoperatively and during the follow-up period (at 4 weeks, 12 weeks, 6 months, and annually) until healing was confirmed (20). We acknowledge the significant value of the Radiographic Union Scale in Tibial fractures (RUST). However, given that this is a retrospective study spanning from 2010 to 2019, the RUST score was not routinely recorded in clinical records at the time of treatment. To minimize subjectivity in the absence of RUST scores, the radiographic assessment was performed independently by two senior orthopedic surgeons who were blinded to the patients’ laboratory results (including fibrinogen levels). Any disagreements regarding the healing status were resolved through consensus or by consulting a third senior surgeon (21).We acknowledge that pooling clavicle and femur fractures may introduce heterogeneity due to differences in anatomical location, biomechanical environment, and healing potential. Unfortunately, detailed fracture classification according to the AO/OTA system and soft tissue injury grading (Tscherne classification for closed fractures or Gustilo-Anderson classification for open fractures) were not routinely documented in the retrospective medical records spanning 2010–2019. Future prospective studies should incorporate these standardized classifications to validate our findings in specific fracture subtypes. This study has been approved by the Clinical Research Ethics Committee of Shandong Provincial Hospital Affiliated to Shandong First Medical University, and the requirement for informed consent was waived because of the retrospective nature of the study. All patients were followed up for at least one year.

### Analytical variables

Patients’ diagnosis and treatment records were collected through the medical record system of Shandong Provincial Hospital Affiliated to Shandong First Medical University, including the patients’ basic information (age, gender, smoking habit, and alcoholism), injury mechanism (traffic accidents, high falls, falls, and other injury mechanisms), fracture characteristics (malformation and mobility), and the American Society of Anesthesiologists (ASA) Classification. Fasting venous blood samples were collected on the day of admission and during hospitalization and sent to the laboratory of Clinical Medical Laboratory Department of the Shandong Provincial hospital Affiliated to Shandong First Medical University for analysis of plasma coagulation examination, prothrombin time (PT), activated partial thromboplastin time (APTT), the plasma fibrinogen concentration (FIB), and the plasma D-dimer concentration (DD). The blood coagulation test was performed using a fully automated blood coagulation instrument (STAGO, France).

Fasting venous blood samples were collected on the day of admission (prior to surgical intervention) for analysis of plasma fibrinogen concentration. We recognize that fibrinogen levels measured at this single time point reflect the acute-phase response to trauma and may not represent longitudinal trends or postoperative values.

### ORIF treatment

**Figure 2:**
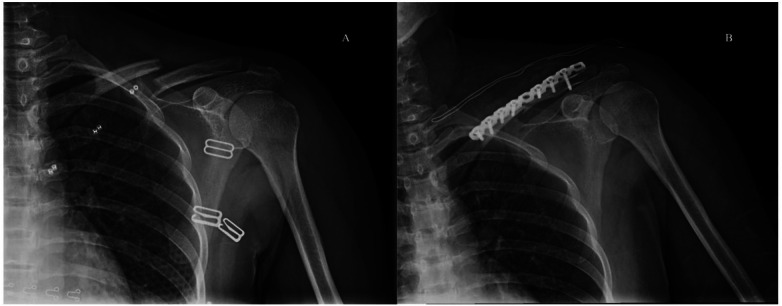
A 32-year-old female patient showing the shoulder joint DR in positive position. **(A)** Discontinuous bone with the broken ends misplaced into angles and the surrounding soft tissue swollen. **(B)** After internal fixation, showing the fracture line blurred, the broken end aligned appropriately, and the internal fixator not broken.

Fractures were generally treated with the open reduction and internal fixation (ORIF), which consisted of three stages: reduction, fixation, and rehabilitation (Figure 2). The skin condition of the patient was evaluated before the operation to prevent postoperative infection and nonhealing of the skin. Weight bearing should be avoided within one year after the operation to prevent loosening of the internal fixation. The fixation can be removed based on either the patient's recovery or patient's desire one year after the operation. The length of hospital accommodation was determined based on the patient's recovering conditions. After discharge, regular x-ray re-examination was performed for one year.

### Statistical analysis

In this study, continuous variables were represented as mean ± standard deviation (SD) or median (interquartile range, IQR), while classified variables were presented as frequency or percentage. Significant differences between the healing and the nonunion groups were assessed using the student's t-test or K-W test for continuous variables and the chi-square test and Fisher's exact test for categorical variables. In univariate analysis, nonunion was used as the dependent variable to evaluate the influence of other variables on the occurrence of nonunion. To investigate the independent effect of FIB on nonunion, Multivariate logistic regression models were constructed under three scenarios: no adjustment (no adjustment for other covariates), adjustment Ⅰ (with adjustment of variables gender and age), and adjustment Ⅱ (with adjustment of variables gender, age, mechanism of injury, and ASA classification). Covariate evaluations and filters were performed to determine the adjusted variables, with our selection criteria as that the change of the risk factor regression coefficient was more than 10% after the covariate was introduced into the basic model. The linear relationship between nonunion and FIB was investigated based on the generalized additive model (GAM) and smoothing curve fitting. All statistical analyses were performed based on the R software (http://www.R-project.org, the R Foundation) and EmpowerStats (http://www.empowerstats.com, X&Y Solutions, Inc., Boston, MA, USA) with a *p*-value less than 0.05 considered to be statistically significant.

Given the limited number of nonunion events (*n* = 23) relative to the number of covariates entered into multivariable models, we acknowledge the risk of overfitting. The adjusted models were constructed with parsimony in mind, and results should be interpreted with caution. We explicitly discuss this limitation in the Discussion section.

## Results

Clinical characteristics analysis of patients with complete healing and nonunion are summarized in Table 1. A total of 338 patients with clavicular or femoral fractures were enrolled, including 315 patients with complete union and 23 patients with nonunion, with an average nonunion rate of 6.8%.Comparative analysis revealed significant differences between complete healing group and nonunion group in terms of FIB (*P* = 0.011) and ASA Classification *(P* = 0.02), while no significant difference was observed between the two groups in other variables (*P* > 0.05).

**Table 1:** Clinical characteristics of fracture patients in healing and nonunion groups.

Characteristics^a^	Healing	Nonunion	*P* value
*N*	315	23	
Age, mean (SD), year	44.59 ± 15.56	44.13 ± 20.21	0.894
PT, mean (SD), s	12.00 ± 1.37	12.42 ± 1.35	0.168
APTT, mean (SD), s	27.71 ± 5.17	29.40 ± 5.88	0.143
FIB, mean (SD), g/L	3.03 ± 0.95	3.59 ± 1.43	0.011*
D-dimer, Median (Q1–Q3), mg/L	0.76 (0.40–1.72)	0.8（0.40–1.07）	0.717
Gender, *N* (%)			0.171
Male	221 (70.16%)	13 (56.52%)	
Female	94 (29.84%)	10 (43.48%)	
ASA classification, *N* (%)			0.02*
Ⅰ & Ⅱ	283 (89.84%)	17 (73.91%)	
Ⅲ & Ⅳ	32 (10.16%)	6 (26.09%)	
Alcoholism, *N* (%)			0.201
Yes	43 (13.65%)	1 (4.35%)	
No	272 (86.35%)	22 (95.65%)	
Smoke, *N* (%)			0.122
Yes	52 (16.51%)	1 (4.35%)	
No	263 (83.49%)	22 (95.65%)	
Mechanism of Injury, *N* (%)			0.085
Fall Injury	188 (59.68%)	8 (34.78%)	
Traffic accident injury	79 (25.08%)	11 (47.83%)	
High Fall Injury	9 (2.86%)	1 (4.35%)	
Other injuries	39 (12.38%)	3 (13.04%)	
Malformation, *N* (%)			0.464
Yes	113 (35.87%)	10 (43.48%)	
No	202 (64.13%)	13 (56.52%)	
Mobility, *N* (%)			0.641
Normal	176 (55.87%)	14 (60.87%)	
Limited	139 (44.13%)	9 (39.13%)	

PT, prothrombin time; APTT, activated partial thromboplastin time; FIB, the plasma fibrinogen concentration; D-dimer, the plasma D-dimer concentration; ASA classification, Alcoholism the American Society of Anesthesiologists.

^a^ Data are presented as number (%), mean ± standard deviation, or median (interquartile range, IQR).

* *P* < 0.05 was statistically significant.

**Figure 3:**
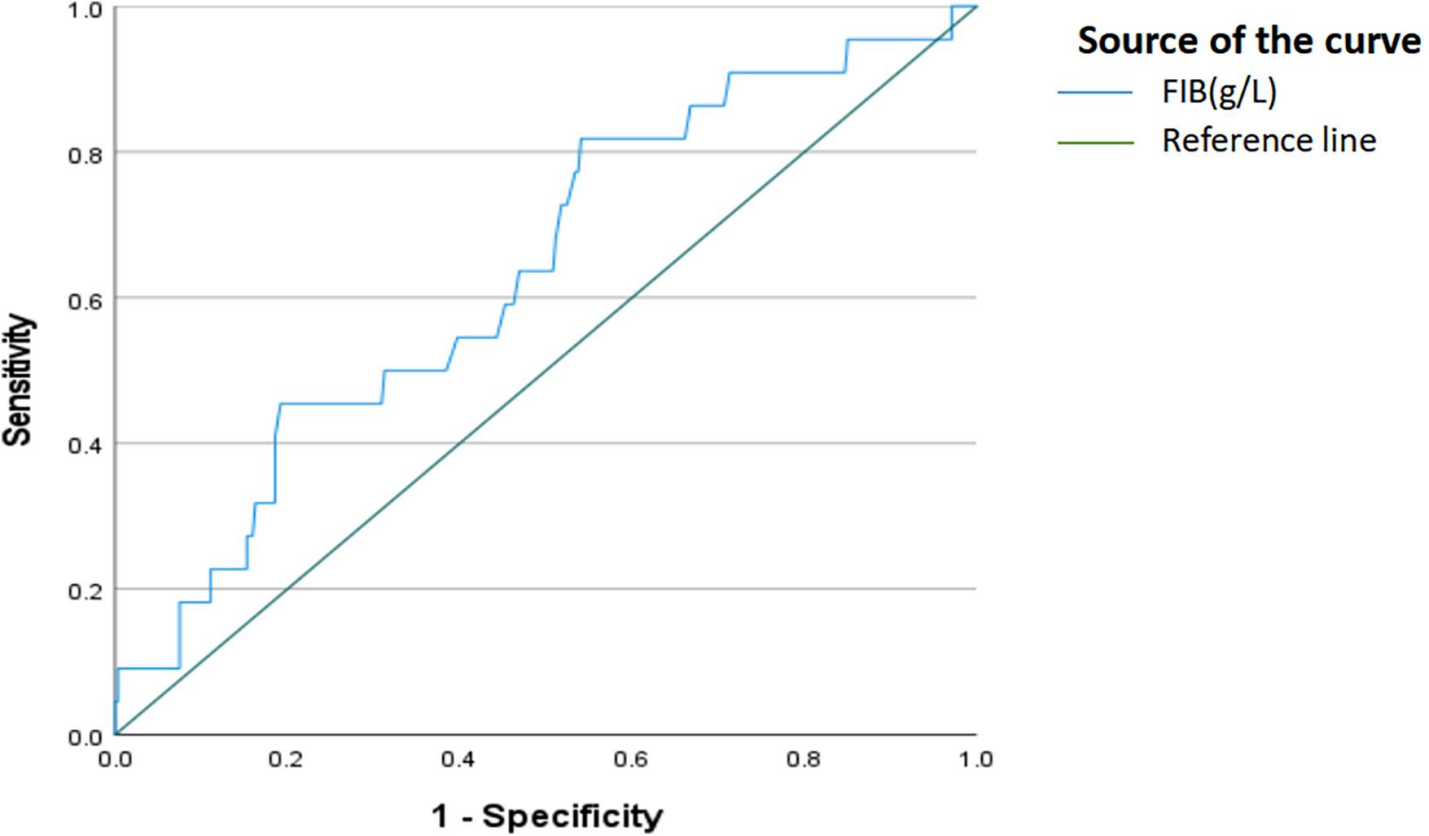
Receiver operating characteristic (ROC) curve for fibrinogen concentration (FIB, g/L) as a predictor of fracture nonunion. The area under the curve (AUC) was 0.635 (95% CI: 0.517-0.752), indicating modest discriminative performance. The optimal cutoff value was 2.795 g/L (sensitivity 81.8%, specificity 45.9%). The diagonal reference line represents no discrimination (AUC = 0.50).

Using ROC curve analysis, the optimal cutoff values for risk factors in predicting nonunion occurrence were determined (Figure 3 and Table 2). FIB demonstrated moderate discriminative ability for fracture nonunion, with an AUC of 0.635 (95% CI: 0.517–0.752), and the optimal cutoff value was identified as 2.795 g/L, yielding a sensitivity of 81.8% and a specificity of 45.9%.

**Table 2:** The predictive power of FIB concentration in nonunion, as demonstrated through ROC curve analysis.

Variable	AUC	95%CI	*P* value	Youden	Optimal cutoff value	Sensitivity	Specificity
FIB(g/L)	0.635	[0.517–0.752]	0.035*	0.277	2.795	81.8%	45.9%

ROC, receiver operating characteristic; FIB, the plasma fibrinogen concentration; AUC, area under the curve.

* *P* < 0.05 was statistically significant.

**Figure 4:**
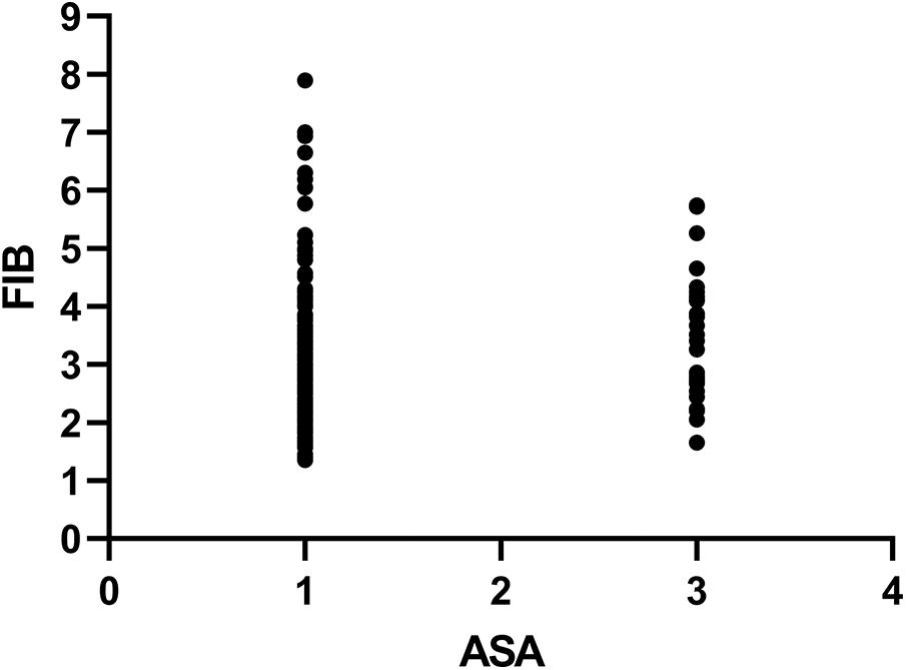
The relationship between pre-operative ASA physical-status grade and plasma fibrinogen concentration. Pearson's r = 0.104, *P* = 0.06.

Visual inspection of the box-plots shows nearly complete overlap across the four ASA categories. Quantitatively, the linear correlation between ASA grade and fibrinogen was weak and non-significant (Pearson's r = 0.104, *P* = 0.06) (Figure 4). These findings indicate that fibrinogen levels are not directly linked to ASA-defined comorbidity burden.

Results of the univariate analysis of the clinical characteristics of fracture patients were given in Table 3. Results showed that the risk of nonunion in patients of traffic accident injury was 3.27 times higher than that of fall injury (*P* = 0.0142), while the FIB was associated with nonunion (OR = 1.55, 95% CI = 1.09–2.20, *P* = 0.0142). The risk of nonunion was increased by 55% with the increase of FIB by 1 g/L. ASA classification was also associated with nonunion (OR =  3.12, 95% CI = 1.15–8.48, *P* = 0.0257). Compared with ASA classification Ⅰ and Ⅱ, the risk of nonunion was increased with ASA classification Ⅲ and Ⅳ. The injury mechanism of traffic accidents was related to nonunion (OR = 3.27, 95% CI = 1.27–8.44, *P* = 0.0142). The risk of nonunion of traffic accident injury was higher than those of fall injury and other injuries.

**Table 3:** Relationship clinical characteristics and nonunion evaluated based on a univariate logistic regression model.

Characteristics^a^	Total	OR	95% CI	*P* value
AGE, mean (SD), years	44.56 ± 15.88	1	(0.97, 1.03)	0.8932
D-dimer, Median (Q1–Q3), mg/L	0.76 (0.4–1.72)	0.97	(0.85, 1.12)	0.7169
PT, mean (SD), s	12.02 ± 1.37	1.19	(0.93, 1.53)	0.1745
APTT, mean (SD), s	27.82 ± 5.22	1.06	(0.98, 1.14)	0.1453
FIB, mean (SD), g/L	3.07 ± 0.99	1.55	(1.09, 2.20)	0.0142*
Gender, *N* (%)				
Male	234 (69.23%)	Ref		
Female	104 (30.77%)	1.81	(0.77, 4.27)	0.1764
ASA classification, N (%)				
Ⅰ & Ⅱ	300 (88.76%)	Ref		
Ⅲ & Ⅳ	38 (11.24%)	3.12	(1.15, 8.48)	0.0257*
Smoke, *N* (%)				
Yes	53 (15.68%)	Ref		
No	285 (84.32%)	4.35	(0.57, 32.98)	0.1549
Alcoholism, *N* (%)				
Yes	44 (13.94%)	Ref		
No	294 (86.06%)	3.48	(0.46,26.47)	0.2287
Mechanism of Injury, *N* (%)				
Fall Injury	196 (57.99%)	Ref		
Traffic accident injury	90 (26.63%)	3.27	(1.27,8.44)	0.0142*
High Fall Injury	10 (2.96%)	2.61	(0.29,23.19)	0.3890
Other injuries	42 (12.43%)	1.81	(0.46,7.12)	0.3973
Malformation, *N* (%)				
Yes	123 (36.39%)	Ref		
No	215 (63.61%)	0.73	(0.31, 1.71)	0.4658
Mobility, *N* (%)				
Normal	190 (56.21%)	Ref		
Limited	148 (43.79%)	0.81	(0.34, 1.94)	0.6415

PT, prothrombin time; APTT, activated partial thromboplastin time; FIB, the plasma fibrinogen concentration; D-dimer, the plasma D-dimer concentration; ASA classification, Alcoholism the American Society of Anesthesiologists; Ref, reference.

^a^ Data are presented as number (%), mean ± standard deviation, or median (interquartile range, IQR).

* *P* < 0.05 was statistically significant.

**Figure 5:**
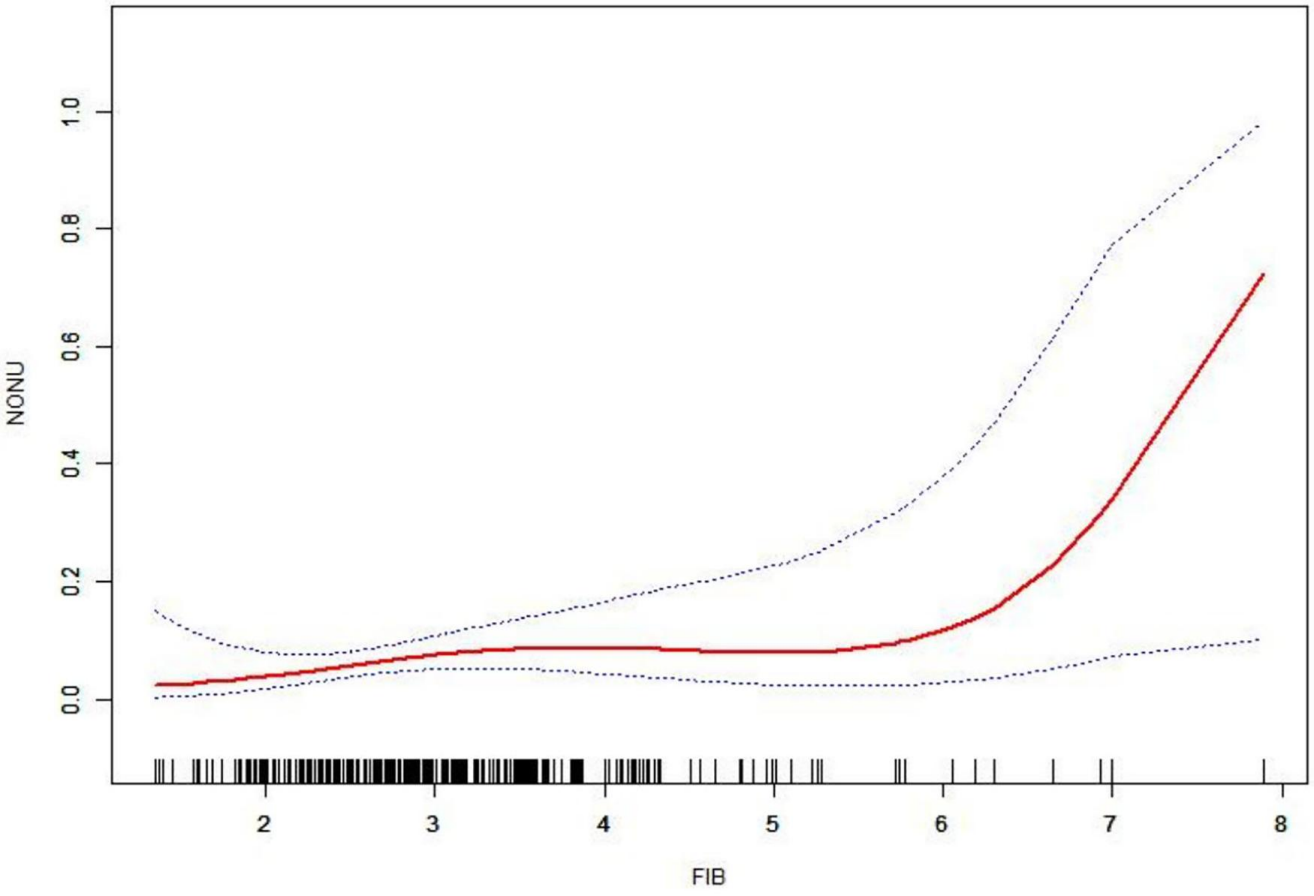
Smoothing curve showing the relationship between fibrinogen concentration (FIB, g/L) and predicted probability of nonunion (NOUN, %). The solid red line represents the fitted curve from generalized additive modeling, with blue dashed lines indicating the 95% confidence interval. The curve suggests a nonlinear, positive association between higher fibrinogen levels and increased nonunion risk, though confidence intervals widen at extreme values due to limited data.

The smoothing curve fitting demonstrated the non-linear relationship between fibrinogen concentration and nonunion (Figure 5). Fibrinogen concentrations increased gradually within the 95% confidence interval, indicating an increased risk of nonunion with increased FIB concentrations.

Results of multivariate logistic regression analysis were given in Table 4. The risk of nonunion increased by 55% (95% CI = 1.09–2.20, *P* = 0.0142) for each unit of fibrinogen increased without adjustment of any variables. When the fibrinogen was adjusted for gender and age, the risk of nonunion increased by 64% for each additional unit of fibrinogen increased (95% CI = 1.14–1.36, *P* = 0.0072). When fibrinogen was adjusted for age, sex, mechanism of injury, and ASA grade, the risk of nonunion increased by 48% for each additional unit of fibrinogen increased (95% CI = 1.03–2.15, *P* = 0.0362).

**Table 4:** Results of multivariate logistic regression analysis used to evaluate the relationship between nonunion and FIB concentration.

Model	FIB (g/L)		
	OR	95% Cl	*P* value
Non-adjusted	1.55	(1.09, 2.20)	0.0142*
Adjusted I	1.64	(1.14, 2.36)	0.0072*
Adjusted II	1.48	(1.03, 2.15)	0.0362*

*P < 0.05 was statistically significant. Non-adjusted model: None variables were adjusted. Adjust I model: two variables (gender and age) were adjusted. Adjust II model: four variables were adjusted, i.e., age, gender, mechanism of injury, and the American Society of Anesthesiologists (ASA) classification.

## Discussion

This study illustrated a significant association between plasma fibrinogen concentration and the incidence of nonunion in fracture patients. Our findings revealed that elevated levels of plasma fibrinogen are independently associated with an increased risk of nonunion, though this association does not imply casusation. Specifically, for every 1 g/L increase in fibrinogen concentration, the risk of nonunion rose significantly by 48% [adjusted odds ratio [OR] = 1.48, 95% confidence interval [CI], although not explicitly reported, supports statistical significance]. This association remained robust after adjusting for mutiple confounding factors, including age, gender, injury mechanism, and ASA classification. These results are consistent with the hypothesis that plasma fibrinogen may be involved in pathways related to bone healing, though alternative explanations including confounding by unmeasured inflammatory factors cannot be excluded.

Plasma fibrinogen serves as the precursor to fibrin, which is essential for blood coagulation. During the final stage of clot formation, soluble fibrinogen is converted into insoluble fibrin to form a stable clot. Therefore, as the basic index of preoperative assessment, the determination of plasma fibrinogen is helpful to evaluate the coagulation status. Studies have shown that fibrinogen can be used as an early predictor of bleeding, even in fractures (22). Studies have shown that as an acute phase reactive protein (APRP), fibrinogen plays a key role in activating and mediating inflammation and is a pathological indicator of many kinds of inflammation, such as acute infection, acute glomerulonephritis, burn, and shock, with the fibrinogen reactivity increased after major surgery (16, 23). The excessive fibrin deposition can slow the wound recovery during the recovery period, while the excessive fibrin can be removed under the action of fibrinolytic enzyme (22, 24).

In this study, we investigated the association between plasma fibrinogen concentration and the nonunion in fracture patients. Our results suggest that plasma fibrinogen concentration is significantly associated with the occurrence of nonunion. We found that fibrinogen plays a certain role in promoting osteogenesis both *in vivo* and *in vitro*. Fibrinogen influences bone formation, and increased fibrinogen concentration may accelerate the healing of bone defects. In terms of clinical characteristics, after adjusting for age, gender, ASA classification, and other confouding factors, we compared healing outcomes among patients and found that the risk of non-healing increased by 48%. Specifically, there were significant differences in FIB (*P* = 0.011) and ASA (*P* = 0.02) between complete healing and nonunion groups (*P* > 0.05). These results were consistent with those reported in the previous studies (25), indicating that the level of fibrin concentration may serve as a diagnostic indicator for patients with complete healing and nonunion of bone. Our study also found that fibrinogen serves as a superior biological marker for diagnosing fracture nonunion compared to white blood cell count, demonstrating the highest accuracy in identifying fracture nonunion. Fibrinogen concentration in healing patients was significantly lower than that in nonunion patients (26). Santos et al. reported both pro-healing and pro-inflammatory effects of fibrinogen (27). In the variable analysis, we adjusted for age, gender, ASA classification, and other covariates when administering fibrinogen levels. By comparing the healing outcomes of fracture patients, we found that an increased concentration of fibrinogen was significantly associated with a higher incidence of nonunion (*P* < 0.05). Correlation analysis showed that bone nonunion was related to not only to patients’ physical condition and injury mechanism but also showed a significant correlation with fibrinogen levels(*P* = 0.0142). These results suggested that the increase of fibrinogen concentration can be used as a potential indicator to predict the occurrence of nonunion. Previous studies have shown that fibrinogen can also be used as a predictor of bleeding, including in fracture patients (22). Multivariate logistic regression analysis further confirmed the association between fibrinogen concentration and nonunion. High fibrinogen concentration is associated with delayed healing of chronic wounds. Fibrinogen exhibits dual pro-inflammatory and anti-inflammatory roles in bone repair, which may influence the healing process. Elevated fibrinogen levels might serve as a biomarker to guide therapeutic interventions, such as anti-inflammatory medications or anticoagulant therapies. Additionally, dynamic monitoring of fibrinogen levels could assist in predicting healing outcomes. Fibrinogen can be conducive to control and help remove some potential infections. Previous studies have shown that fibrinogen can recruit related cells to Promote fracture healing. Specifically, fibrin can capture bacteria in the damaged part of tissue to prevent infection in the injured tissue. In fracture, the inflammatory cytokines released by necrosis tissue can play a common role with integrin on fibrin in the site of fracture and plays a crucial role in fracture healing (28). Fibrinolysis is an important factor in fracture healing, and with the deposition of fibrin matrix, the probability of nonunion increases (29). In the initial stage following fracture, FIB levels exhibit a specific pattern of change. At this point, FIB plays a crucial role in initiating the body's response to the fracture. It helps to stabilize the injured area by promoting the formation of a blood clot around the fracture site. This blood clot acts as a protective framework, preventing further damage to surrounding tissues and providing a foundation for subsequent repair processes. As the fracture progresses into the inflammatory phase, FIB levels continue to fluctuate. During this phase, FIB is actively involved in the inflammatory response. It aids in attracting immune cells to the site of injury, facilitating the clearance of debris and the removal of damaged tissue. By doing so, FIB contributes to creating a favorable environment for tissue repair and regeneration. In the proliferative phase of fracture healing, FIB takes on a different set of functions. Its levels are adjusted to support the growth of new bone tissue. FIB provides structural support for the developing callus, helping to maintain the integrity of the forming bone matrix. It also plays a role in the recruitment and differentiation of osteoblasts, the cells responsible for bone formation. This ensures that the newly formed bone is properly organized and has the necessary strength to withstand mechanical stress.Finally, in the remodeling phase, FIB levels stabilize and adapt to the ongoing maturation of the bone. FIB continues to assist in the fine-tuning of bone structure. It helps to reshape the callus into a more normal bone structure by influencing the activity of osteoclasts, which are responsible for bone resorption. This balanced interaction between FIB and bone- remodeling cells ensures that the fracture site regains its original strength and functionality. The changes in FIB levels during the above four stages and their corresponding roles demonstrate the importance of FIB as a dynamic component of the body's response to bone injury and its significance in the overall recovery process (17).Our results showed that as the risk of nonunion increased as the fibrinogen concentration increased. These results are consistent with previous findings, demonstrating that increased fibrinogen inhibits fracture repair (16). Therefore, we conclude that the plasma fibrinogen concentration is statistically associated with the occurrence of nonunion in this cohort.

We observed only a weak and non-significant correlation between preoperative ASA class and plasma fibrinogen levels (Pearson's r = 0.104, *P* = 0.06). This suggests that fibrinogen is unlikely to serve merely as a surrogate marker for the overall comorbidity burden reflected by the ASA score. Several non-mutually exclusive interpretations warrant consideration. First, fibrinogen is an acute-phase reactant primarily regulated by IL−6-mediated hepatic transcription, whereas the ASA classification emphasizes chronic end-organ dysfunction. This temporal mismatch between acute inflammatory responses and long-standing comorbidities may attenuate any linear correlation. Second, genetic variability—such as polymorphisms in FGA and FGB—accounts for up to 40% of baseline fibrinogen variation and operates independently of comorbid conditions. Third, localized rather than systemic stimuli—such as tissue hypoxia at the surgical site—may drive fibrinogen production, further obscuring a direct ASA–fibrinogen association. Whether fibrinogen functions as an independent predictor of adverse outcomes or simply represents an epiphenomenon of underlying inflammation remains uncertain. On one hand, elevated fibrinogen promotes erythrocyte aggregation, increases blood viscosity, and facilitates microvascular thrombosis, providing a biologically plausible mechanism contributing to organ dysfunction. On the other hand, residual confounding due to unmeasured inflammatory sources—such as subclinical infection or undiagnosed malignancy—could artificially inflate its apparent predictive value. Until interventional studies demonstrate that reducing fibrinogen levels leads to improved clinical outcomes, a causal role cannot be definitively established. Future research should therefore incorporate repeated perioperative measurements to characterize temporal patterns, apply Mendelian randomization approaches to reduce confounding, and investigate post-translationally modified fibrinogen isoforms—such as glycated or oxidized forms—that may exhibit distinct biological activities. Greater granularity in these areas will help determine whether fibrinogen is a modifiable risk factor or merely an inflammatory “bystander,” thereby guiding the development of targeted therapeutic strategies.

Several limitations warrant careful consideration. First, the small number of nonunion events (*n* = 23) relative to the number of covariates in multivariable models raises concerns about overfitting and model stability. The adjusted odds ratios should be interpreted as exploratory rather than definitive estimates of effect size. Validation in larger cohorts is essential. Second, the modest AUC of 0.635 indicates that fibrinogen alone has limited discriminative ability for predicting nonunion. While statistically significant, this level of performance suggests that fibrinogen should be considered as a complementary biomarker rather than a standalone diagnostic tool. The clinical utility may lie in risk stratification when combined with other clinical factors, rather than in isolation. Third, as a retrospective observational study, we cannot infer causality from the observed associations. Elevated fibrinogen may represent: (a) a causal mediator in the pathophysiology of impaired bone healing; (b) a risk marker reflecting underlying inflammatory or thrombotic propensity; or (c) an epiphenomenon of the acute-phase response to trauma. The biological mechanisms remain hypothetical, and interventional studies would be required to establish causality. Fourth, fibrinogen was measured only at admission, reflecting the acute-phase response to initial trauma. Perioperative fluctuations, longitudinal trends, and postoperative values were not captured. Acute trauma and surgical intervention themselves influence fibrinogen levels, and our results may not apply to serial measurements or values obtained at different time points. Whether admission fibrinogen predicts nonunion risk beyond its role as an acute-phase reactant requires prospective validation with repeated measurements. Fifth, the pooling of clavicle and femur fractures introduces heterogeneity, as these anatomical sites differ in blood supply, mechanical loading, and healing potential. The lack of AO/OTA fracture classification and standardized soft tissue injury grading limits the generalizability of our findings to specific fracture patterns. Residual confounding from unmeasured injury severity factors cannot be excluded. Finally, we excluded patients with active infections and hematological malignancies, limiting the applicability of our findings to these high-risk populations.

## Conclusion

Our study revealed that the plasma fibrinogen levels in patients with nonunion were significantly higher compared to those with complete healing. Additionally, the risk of nonunion increased by 48% with the increase of FIB by every 1 g/L. Our findings provide an important diagnosis tool for clinicians to identify patients with high risk of nonunion.

## Data Availability

The original contributions presented in the study are included in the article/Supplementary Material, further inquiries can be directed to the corresponding author.
